# Concentration, Origin and Health Hazard from Fine Particle-Bound PAH at Three Characteristic Sites in Southern Poland

**DOI:** 10.1007/s00128-013-1060-1

**Published:** 2013-07-23

**Authors:** Wioletta Rogula-Kozłowska, Barbara Kozielska, Krzysztof Klejnowski

**Affiliations:** 1 Institute of Environmental Engineering, Polish Academy of Sciences, 34 M. Skłodowska-Curie St., 41-819 Zabrze, Poland; 2 Department of Air Protection, Silesian University of Technology, 2 Akademicka St., 44-100 Gliwice, Poland

**Keywords:** PM_1_, PM_2.5_, Carcinogenicity, Diagnostic ratios, Toxicity equivalence factor TEF, Minimum mutagenic concentration MMC

## Abstract

Suspended particles with the aerodynamic diameters not greater than 2.5 μm (PM_2.5_) and 1 μm (PM_1_, sub-fraction of PM_2.5_) were sampled at three sites: an urban background site, rural background site, and urban traffic site in southern Poland. In total, there were 240 samples taken within 02.08.2009–27.12.2010. Fifteen polycyclic aromatic hydrocarbons (PAH) were determined in each dust fraction. The averages of the concentration of total PAH (ΣPAH) and of particular PAH, as well as the share of carcinogenic PAH in total PAH (ΣPAH_carc_/ΣPAH), carcinogenic equivalent, mutagenic equivalent, and TCDD-toxic equivalent appeared high compared to other areas in the world. Their high values express the significance of health hazard from PM and PM-bound PAH in southern Poland. The diagnostic ratios suggest that PM-bound PAH originate from municipal (PM_1−2.5_) and vehicular (PM_1_) combustion.

The adverse health effects of ambient particulate matter (PM) manifest themselves everywhere in the world, especially in heavily polluted regions like southern Poland. The origin, concentrations and health effects of polycyclic aromatic hydrocarbons (PAH) associated with PM, most often with PM_2.5_ and PM_10_ (PM particles with the aerodynamic diameter not greater than 2.5 and 10 μm), have been investigated world-wide at a variety of sites.

Majority of PM-bound PAH is to be found in PM_2.5_ (e.g. Makkonen et al. 2010), usually the core part of PM in urbanized areas, such as Upper Silesia (Rogula-Kozłowska et al. 2012). PM_2.5_, especially its sub-fraction PM_1_, consists of inhalable particles that contribute to formation of active oxides in lungs (de Kok et al. 2006) and are the most cytotoxic ambient particles (Massolo et al. 2002). However, the available data on ambient PAH concentrations in many regions, including southern Poland, are not sufficient to fully assess the PAH effects on human health.

The goal of the presented work was to investigate the ambient concentrations of fifteen PM_1_- and PM_2.5_-bound PAH (Acy, Ace, F, Ph, An, Fl, Py, BaA, Ch, BbF, BkF, BaP, DBA, BghiP, IP)^1^ at three sites, each representing one specific situation in air pollution in southern Poland. Probable sources of PAH and the level of the hazard to humans from the mixture of PAH (i.e. equivalents: carcinogenic equivalent, CEQ, and mutagenic equivalent, MEQ) are also presented.

## Materials and Methods

Diurnal samples of PM_1_ and PM_2.5_ were collected at three sites in southern Poland: urban background (UB) and urban traffic (UT, urban site directly affected by road traffic) sites in Katowice, and a regional background site (RB) in Złoty Potok. The samples were collected with the use of a high volume sampler (Digitel DHA-80) and, respectively, PM_1_ and PM_2.5_ measuring heads onto Whatman quartz fiber filters (QMA).

The UB measuring point in Katowice (50º15′56″N, 18º58′40″E, 274 m a.s.l.), was located in the western part of the city, in a residential district, about 3.2 km west of the city center. There were blocs of flats, commercial areas, and a railway line in its neighborhood; a post-mining terrain is some distance off.

The UT measuring point in Katowice (50º14′49″N, 19º01′04″E, 298 m a.s.l.), was located near the A4 highway, almost on the shoulder, about 1.5 km south of the city center. The volume of traffic is about 30,000 vehicles per day at this point.

The RB measuring point (50º42′59″N, 19º26′37″E, 283 m a.s.l.), was in Złoty Potok (commune of Janów), approximately 20 km south-east of Częstochowa and 25 km north of Zawiercie. It was surrounded by meadows and arable lands. Several chalets and a forester’s house, all heated with wood, were about 150 m away.

Within 02.08.2009–27.12.2010, in each, heating and non-heating, season two measurement campaigns were held for each fraction at each point, no two consecutive campaigns at the same point. From 6 to 14 (usually 10) diurnal samples of one dust fraction (PM_1_ or PM_2.5_) were taken during a campaign. Forty samples of each PM_1_ and PM_2.5_ were taken at each point (240 samples in total).

The mass of the sampled dust was determined gravimetrically (Sartorius balance, resolution 0.01 g) according to the CSN EN 14907 standard (Ambient air quality – Standard gravimetric measurement method for the determination of the PM_2.5_ mass fraction of suspended PM). Before each weighing, the filters were conditioned for at least 48 h at the air temperature of 20 ± 1°C and air relative humidity of 50 % ± 5 % in the weighing room.

The dust was extracted from filters with dichloromethane (CH_2_Cl_2_) in an ultrasonic bath. The extract was filtered, rinsed and dried in helium, dissolved in 2-propanol (limpid solution) and diluted with re-distilled water (propanol: water proportion of 15:85 V/V). The solid phase of the samples was selectively purified by extracting (SPE) in C-18 columns filled with octadecylsilane. The columns were placed in an extraction manifold and conditioned by rinsing with methanol, then with mixture of 2-propanol and re-distilled water (15:85 V/V); they were not allowed to dry.

Directly before the extraction, each column was rinsed with 5 cm^3^ of the 2-propanol and water mixture (15:85 V/V). The samples were passed through the columns under vacuum. Then they were washed with the mixture of 2-propanol and water (15:85 V/V) and dried at low vacuum. The PAH fraction was eluted with dichloromethane. The extract was condensed in helium to the volume of 0.5 cm^3^. The product was analyzed chromatographically.

A Clarus 500 Perkin Elmer gas chromatograph with a flame ionization detector (FID) was used. The sample components were separated with the use of a Restek RTX-5 capillary column (30 m × 0.32 mm, film thickness of 0.25 μm). The flow rate of the carrier gas, helium, was 1.5 cm^3^/min. The calibration curves for the quantitative analysis were prepared for 15 standard PAH. The linear correlation between the peak surface areas and the PAH concentrations was checked within the range 1–20 μg/mL (correlation coefficients 0.99, PAH Mix PM-611 Ultra Scientific standard at the concentration 100 μg/mL of each PAH in dichloromethane).

The samples were introduced onto the column directly or with a split/splitless injector. The evaporator temperature was 240°C, the detector one–280°C. The temperature on the column grew at 10°C/min from 4 min-lasting 60°C, to 14 min-lasting 280°C. The entire analysis time was 40 min. FID was fed with hydrogen (45 cm^3^/min), air (450 cm^3^/min) and helium (30 cm^3^/min).

The analysis of each campaign sample-series was accompanied by the analysis of a blank sample, which consisted in applying the whole analytical procedure to a clean quartz fiber filter. A blank result was used to adjust the PAH concentration only if the blank exceeded 10 % of the PAH concentration. The detection limits (DL), received from the statistical development of the blank results (10–11 blanks for each PAH, PN-EN 15549 standard), were between 0.01 and 0.03 ng/m^3^ (average air flow rate 700 m^3^/24 h).

The performance of the applied method was verified by analyzing the NIST SRM 1649b reference material and comparing the results with the certified concentrations of the investigated PAH (analyzes of 3 certified samples of dust, 0.18 g each). The standard recovery was from 92 % to 111 % (Table 1).

**Table 1:** Average concentrations of 15 PAH in three samples of SRM 1649b

	Average content of individual compound in mass of dust (mg/kg)		Recovery (%)
	SRM 1649b	Determined	
Ace	0.192	0.190	99
Acy	0.184	0.200	111
F	0.222	0.210	93
Ph	3.941	3.630	92
An	0.403	0.390	97
Fl	6.140	6.360	104
Py	4.784	4.990	104
BaA	2.092	2.110	101
Ch	3.008	2.910	97
BbF	5.990	5.790	97
BkF	1.748	1.680	96
BaP	2.470	2.310	94
IP	2.960	2.900	98
DBA	0.290	0.280	97
BghiP	3.937	3.970	101

The cumulative health hazard from a mixture of PAH is expressed quantitatively as the CEQ or mutagenic MEQ relative to the carcinogenicity or mutagenicity of BaP, respectively, or as the TCDD-toxic equivalent (TEQ) relative to the 2,3,7,8-tetrachlorodibenzo-*p*-dioxin toxicity. For each site and, separately, for PM_1_ and PM_2.5_, CEQ, MEQ, and TEQ were computed as linear combinations of the concentrations of PM-bound PAH and: their toxicity equivalence factors TEF (CEQ, Eq. 1), or their PAH minimum mutagenic concentrations MMC (MEQ, Eq. 2), or their TCDD-TEF (TEQ, Eq. 3). The values of TEF, MMC and TCDD-TEF for the particular PAH, used in Eqs. 1–3, are taken from Nisbet and LaGoy (1992), Durant et al. (1996), and Willett et al. (1997).

From Eq. 4 the proportion was computed of the sum of the concentrations of the carcinogenic PAH (ΣPAH_carc_) to the sum of the concentrations of the 15 determined PAH (ΣPAH). The closer the value of ΣPAH_carc_/ΣPAH to 1 is, the more hazardous ΣPAH is to humans.

1 $$ \begin{aligned} {\text{CEQ}} &  = 0.001 \times \left( {[{\text{Acy]}} + [ {\text{Ace]}} + [{\text{Fl}}] + [{\text{Ph}}] + [{\text{Fla}}] + [{\text{Py}}]} \right) + 0.01 \times \left( {[{\text{An}}] + [{\text{Ch]}} + [{\text{BghiP}}]} \right) \\ & \quad + 0.1 \times \left( {[{\text{BaA}}] + [{\text{BbF}}] + [{\text{BkF}}] + [{\text{IP}}]} \right) + 1 \times [{\text{BaP}}] + 5 \times [{\text{DBA}}] \, \\ \end{aligned} $$

2 $$ \begin{aligned} {\text{MEQ}} &  =  0.00056 \times [{\text{Acy}}] + 0.082 \times [{\text{BaA}}] + 0.017 \times [{\text{Ch}}] + 0.25 \times [{\text{BbF}}] + 0.11 \times [{\text{BkF}}] \\ & \quad + 1 \times [{\text{BaP}}] + 0.31 \times [{\text{IP}}] + 0.29 \times [{\text{DBA}}] + 0.19 \times [{\text{BghiP}}] \\ \end{aligned} $$

3 $$ \begin{aligned} {\text{TEQ}} & = 0.000025 \times [{\text{BaA}}] + 0.00020 \times [{\text{Ch}}] + 0.000354 \times [{\text{BaP}}] + 0.00110 \times [{\text{IP}}] \\ & \quad + 0.00203 \times [{\text{DBA}}] + 0.00253 \times [{\text{BbF}}] + 0.00487 \times [{\text{BkF}}] \\ \end{aligned} $$

4 $$ \Upsigma {\text{PAH}}_{\text{carc}} /\Upsigma {\text{PAH}} =
\left( {[{\text{BaA}}] + [{\text{BaP}}] + [{\text{BbF}}] +
[{\text{BkF}}] + [{\text{Ch}}] + [{\text{DBA}}] + [{\text{IP}}]}
\right) {\text{/}} \left( {[\Upsigma {\text{PAH}}]} \right) $$

## Results and Discussion

**Fig. 1:**
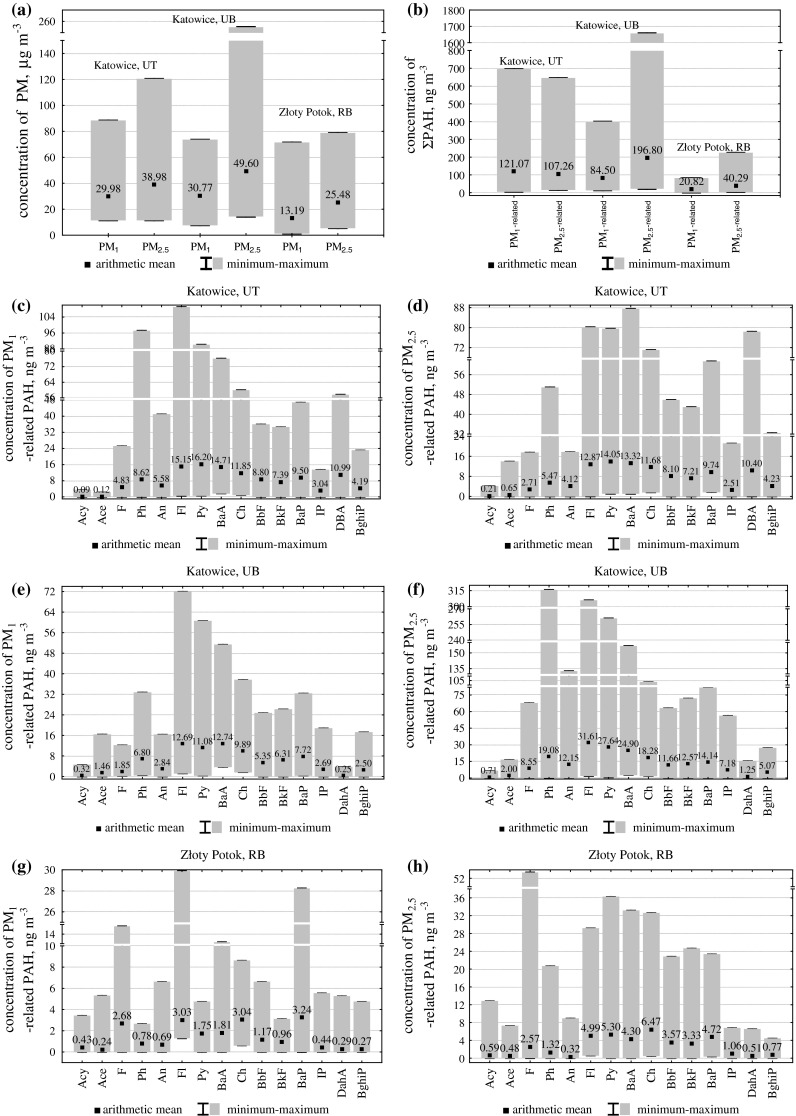
The ranges of diurnal concentrations and the values of averages over the measuring period of PM **a**, ΣPAH **b** and the fifteen PAH **c**–**h** at three sites in southern Poland

The ambient concentrations of PM in the cities of southern Poland are among the highest in Europe (Rogula-Kozłowska et al. 2012). The PM_1_ and PM_2.5_ concentrations at the selected sites were also very high during the whole measuring period. The lowest concentrations of both fractions, diurnal and averaged over the whole measuring period, occurred at RB. But even at RB, beyond any effects of strong PM_2.5_ sources, the average PM_2.5_ concentration was greater than 25 μg/m^3^, the standard for PM_2.5_ (Fig. 1).

Like elsewhere in southern Poland (Rogula-Kozłowska et al. 2012), the highest diurnal PM concentrations of the heating season had a decisive effect on the average concentrations of both PM_1_ and PM_2.5_ at the three sites. The differences in the diurnal concentrations between heating and non-heating seasons caused very wide diurnal concentration ranges for each site and dust fraction (Fig. 1).

The measuring period average PM_2.5_ concentration at UT, the point directly affected by vehicular emission, but not by municipal sources, was lower than at UB. The average PM_1_ concentrations at both sites were close. It means that the average PM_1_ contribution to PM_2.5_ was greater at UT than at UB (average PM_1_/PM_2.5_ at UT and UB were 0.75 and 0.62, respectively). The range of the diurnal PM_1_ concentrations was also wider at UT than at UB; their minimum at UT was relatively high – 11.8 μg/m^3^. Instead, the range of the diurnal PM_2.5_ concentrations was greater at UB. This suggests significant effects of local stationary sources on PM_1−2.5_ and of vehicular sources on PM_1_ concentrations in Katowice.

Like the diurnal concentrations of PM_1_ and PM_2.5_, the diurnal concentrations of PM-bound ΣPAH and all particular PAH also fell within a wide range and their greatest values occurred in the heating season at all sampling sites.

The most obvious cause of such a seasonal variability in the PM and PM-bound PAH concentrations and of the wide ranges of their values is the intensification of the energy production in cold season (hard coal combustion in domestic furnaces) being important, if not the most important, source of PM in southern Poland. Additionally, the high concentrations of air pollutants, including PM, in the lower layer of troposphere and the PAH condensation on PM particles are favored by the weather conditions in winter (shallow mixing layer, low air temperature). In summer, higher air temperature and insolation intensify photochemical decomposition and desorption (evaporation) of PAH from particles. The average ambient concentrations of PM-bound ΣPAH and most of PAH were high at all measuring sites during the whole measuring period. At RB they were higher (sometimes 10 times) than in similar areas not affected by vehicular or industrial emission, such as Virolahti in Finland (regional background, Makkonen et al. 2010). The average PM-bound ΣPAH and BaP concentrations at UB (Katowice) were higher than in other European cities: e.g. Oporto (Portugal; Slezakova et al. 2011), or Zonguldak (Turkey; Akyüz and Çabuk 2008). The diurnal PM_2.5_-bound BaP concentrations reached 80 ng/m^3^ – such high PM-bound BaP concentrations beyond southern Poland occur only in densely populated and highly industrialized regions of Asiatic countries, such as Fushun (residential-commercial site, Kong et al. 2010).

Like the average and diurnal concentrations of PM_1_, the average and diurnal concentrations of PM_1_-bound ΣPAH and all four-, five-, and six-ring PAH were lowest at RB and highest at UT (Fig. 1). The concentrations of PM_2.5_-bound ΣPAH and particular PAH, except DBA and all six-ring PAH, were higher at UB than at UT – the difference may be due to the PM_1−2.5_-bound PAH adsorbed onto soot agglomerates from municipal emission.

At each site, the individual PM_1_-bound three-ring PAH average concentrations were similar, although sometimes higher at RB. However, most of ambient three-ring PAH can occur in gaseous phase, so their PM-bound concentrations may be compared only carefully.

High concentrations of DBA, BghiP and IP are indicative of vehicular emission – six-ring PAH occur in the air in road tunnels and exhaust gases from gasoline engines (Khalili et al. 1995). Their high concentrations at UT and significant linear correlations between the concentrations of IP, DBA, BghiP and ΣPAH (the pairwise computed correlation coefficients R for IP, DBA, BghiP and ΣPAH concentrations, PM_1_- and PM_2.5_-bound ones together, were between 0.81 and 0.93) suggest the dominance of vehicular emissions. At UB, the correlations between these PAH concentrations were weaker, although between some of them significant (R between 0.61 and 0.88); at RB they were weakest (R between 0.37 and 0.61).

The origin of PM at the three sites may be explained by using the molecular diagnostic ratios (Table 2). The proportion [Ph]/([Ph] + [An]) for PM_1_-bound PAH indicates predominance of vehicular emission at UB (Khalili et al. 1995), for PM_2.5_-bound PAH – coal combustion. Similarly, [Ph]/[An], significantly lower for PM_2.5_-bound than for PM_1_-bound PAH, suggests vehicular emission for PM_1_ and PM_1_-bound PAH, and municipal emission, i.e. coal combustion, for PM_2.5_ and PM_2.5_-bound PAH (Rogge et al. 1993a, b). The proportion [Fl]/([Py] + [Fl]) indicates prevailing effect of traffic for PM_1_ and combustion of coal, wood, and natural gas for PM_2.5_ (Ravindra et al. 2006, 2008). [BbF]/[BkF] (Masclet et al. 1987) and [BaP]/[BghiP] (Simcik et al. 1999), although close for PM_1_ and PM_2.5_, suggest municipal emission (coal combustion) as the major source at UB.

**Table 2:** Measuring period averages of selected diagnostic ratios for PM_1_ and PM_2.5_ at three sites in southern Poland

	PM_1_			PM_2.5_		
	Katowice, UT	Katowice, UB	Złoty Potok, RB	Katowice, UT	Katowice, UB	Złoty Potok, RB
[BaA]/[BaP]	2.03	2.78	0.83	1.65	2.33	1.35
[Fl]/([Py] + [Fl])	0.28	0.23	0.55	0.26	0.34	0.29
[BaA]/([Ch] + [BaA])	0.60	0.62	0.27	0.55	0.60	0.37
[BaA]/[Ch]	1.96	2.11	0.68	1.67	1.76	0.71
[BaP]/([BaP] + [Ch])	0.46	0.43	0.46	0.49	0.47	0.40
[BbF]/[BkF]	1.34	0.80	1.38	2.74	1.01	1.14
[Py]/[BaP]	1.39	1.41	1.05	1.51	1.64	1.38
[Ph]/([Ph] + [An])	0.55	0.46	0.65	0.43	0.73	0.89
[Ph]/[An]	1.44	3.82	1.08	1.11	1.83	4.91
[BaP]/[BghiP]	2.71	3.46	2.08	2.11	3.16	4.39
[IP]/[BghiP]	1.14	1.45	1.27	0.51	1.30	1.60
[IP]/([BghiP] + [IP])	0.58	0.75	0.71	0.42	0.69	0.58

Among [Ph]/([Ph] + [An]), [BaP]/([BaP] + [Ch]), [Ph]/[An], [IP]/([BghiP] + [IP]), [Fl]/([Py] + [Fl]), reflecting high contribution of vehicular emission to PM-bound PAH at UT (Table 2), [Fl]/([Py] + [Fl]) and [Ph]/([Ph] + [An]) confirm the contribution of gasoline engines (Khalili et al. 1995; Ravindra et al. 2006, 2008), and [IP]/([BghiP] + [IP]), [Py]/[BaP] – diesel engines (e.g. Oda et al. 2001).

[BaP]/([BaP] + [Ch]), [Py]/[BaP], [IP]/([BghiP] + [IP]) for PM_1_-bound PAH suggest diesel engines as the main PM_1_ source at UB (Rogge et al. 1993a, b; Khalili et al. 1995). Like at UB, some effect of municipal sources is suggested by [BaP]/[BghiP], [BaA]/([Ch] + [BaA]) at UT (Table 2, Sicre et al. 1987; Simcik et al. 1999). At both UT and UB, PM_1_ and PM_1_-bound PAH come from traffic, but the values of [BbF]/[BkF] suggest stationary combustion as a probable source of PM_2.5_ and PM_2.5_-bound PAH (Masclet et al. 1987; Dickhut et al. 2000).

At RB ([BaA]/[BaP], [Fl]/([Py] + [Fl]), [BaA]/([Ch] + [BaA]), [BaA]/[Ch], [Ph]/([Ph] + [An]), [IP]/[BghiP], and [BaP]/[BghiP] allow to relate PM (both fractions) and PM-bound PAH with combustion of wood, coal, and natural gas (Masclet et al. 1987; Sicre et al. 1987; Khalili et al. 1995; Simcik et al. 1999; Dickhut et al. 2000; Ravindra et al. 2008; Kong et al. 2010).

The proportion of the concentration of the sum of four-ring PAH (Fl, Py, BaA, Ch) to the concentration of the sum of five- and six-ring PAH (BbF, BkF, BaP, DBA, BghiP, IP) allows to distinguish between PAH from local emission and PAH brought by long-range transport (Wang et al. 2008). At RB, the mean of this ratio was 2.49 and 3.00 for PM_1_ and PM_2.5_, respectively, at UB – 1.99 and 1.61, at UT – 2.1 and 1.85. At all three sites, but most clearly at RB, the ambient PAH concentrations may be attributed to the pollutants brought from distant regions. The local sources exert probably the greatest effect on the PAH concentrations at UB.

The averages of diurnal ΣPAH_carc_/ΣPAH for PM_1_- and PM_2.5_-bound PAH at all sites were between 0.5 and 0.6. The diurnal values at all sites were also close (Table 3). Consequently, in southern Poland, independently of a measuring site, the contribution of carcinogenic PAH to the sum of 16 so called “EPA priority PAHs” (without naphthalene) is from a little above 10 to even 84 %; it is about 60 % in average. The highest average CEQ, 68.11 ng/m^3^, occurred at UT for PM_1_; the average CEQ for PM_2.5_ was 65.10 ng/m^3^ at UT. Although the ΣPAH concentrations at UB and UT did not differ much the averages of CEQ at UB were lower than at UT: for PM_1_-bound PAH 6 times, and for PM_2.5_-bound PAH 2.5 times. The ranges of the diurnal CEQ for both PM_1_-bound PAH and PM_2.5_-bound PAH at UB were narrower than at UT. Such high as at UT average CEQ were noted by Kong et al. (2010) in Liaoning Province (China) at an industrial/commercial site for PM_2.5_-bound PAH (66.97 ng/m^3^). The values occurring at UT are comparable with those noted by Kong et al. (2010) or Akyüz and Çabuk 2008 (Zonguldak, Turkey) at UB sites.

**Table 3:** The ranges of diurnal values (first column) and the values of averages over the measuring period (second column) of the contribution of carcinogenic PAH to ΣPAH (ΣPAH_carc_/ΣPAH), carcinogenic equivalents (CEQ), mutagenic equivalents (MEQ) and TCDD-toxic equivalent (TEQ) for PM_1_ and PM_2.5_ at three sites in southern Poland

	Katowice, UT		Katowice, UB		Złoty Potok, RB	
PM_1_						
ΣPAH_carc_/ΣPAH	0.16–0.83	0.59	0.28–0.78	0.58	0.22–0.84	0.51
CEQ, ng/m^3^	0.63–310.40	68.11	1.38–51.93	11.87	0.01–30.33	5.17
MEQ, ng/m^3^	0.57–89.13	18.85	1.29–55.21	12.35	0.13–30.79	4.58
TEQ, pg/m^3^	0.21–414.12	87.65	3.23–230.19	52.83	0.16–42.55	10.58
PM_2.5_						
ΣPAH_carc_/ΣPAH	0.19–0.82	0.61	0.14–0.70	0.51	0.21–0.83	0.63
CEQ, ng/m^3^	2.08–474.24	65.10	0.95–195.86	26.47	0.70–37.62	8.59
MEQ, ng/m^3^	2.03–121.36	18.45	0.62–145.56	24.35	1.21–43.04	8.05
TEQ, pg/m^3^	1.37–566.37	88.38	0.66–658.50	110.78	0.88–202.74	30.64

The measuring period average (5.17 and 8.59 ng/m^3^ for PM_1_ and PM_2.5_, respectively) and diurnal CEQ were lower at RB than at UB and UT (Table 3).

The averages of diurnal MEQ at UT and UB were 18.85 and 12.25 ng/m^3^ for PM_1_ and 18.45 and 24.35 ng/m^3^ for PM_2.5_. The measuring period average TEQ for PM_1_-bound PAH at UB (52.83 pg/m^3^) was lower than at UT (87.65 pg/m^3^). For PM_2.5_-bound PAH it was higher at UB than at UT; its diurnal value range was also wider at UB than at UT.

The diurnal and the measuring period averages of MEQ, TEQ and CEQ were lower at RB (Złoty Potok) than at UT and UB (Katowice, Table 3).

Among the three sites, the rural site in Złoty Potok is indicated by (ΣPAH_carc_/ΣPAH), and CEQ, MEQ, TEQ as the one of the least negative effect of PM_2.5_ and PM_2.5_-bound PAH on humans. Not only those coefficients but also the concentrations of PM_2.5_, ΣPAH, and of the majority of particular PAH are lowest at RB. In Złoty Potok, PM_2.5_ and PM_2.5_-bound PAH come from local domestic heating and transport from other regions of southern Poland.

Unlike ones in Złoty Potok, the concentrations of PM_1_ and PM_1_-bound PAH and all the health hazard coefficients at UT by the highway, and the concentrations of PM_2.5_ and PM_2.5_-bound ΣPAH and MEQ and TEQ at UB near the Katowice center, suggest a serious air quality problem causing the threat to human health. At UT, PM_1_ and PM_1_-bound PAH come from road traffic. At UB, PM_1_ comes from road traffic and PM_1−2.5_ from municipal sources; they sum up to PM_2.5_. It seams that in contrast to other parts of Europe, in big cities of southern Poland the hazard from fine dust and dust-bound PAH occurs not only in the areas with the prevailing effect of road traffic but it may be equally high at the sites where the municipal emission prevails. It may occur especially in city centers where compact building arrangement and poor aeration suppress the pollutant transport.

## Footnotes

1 Three-ring PAHs: acenaphthylene (Acy), acenaphthene (Ace), fluorene (F), phenanthrene (Ph), anthracene (An), four-ring PAHs: fluoranthene (F1), pyrene (Py), benzo[a]anthracene (BaA), chrysene (Ch), five-ring PAHs: benzo[b]fluoranthene (BbF), benzo[k]fluoranthene (BkF), benzo[a]pyrene (BaP), dibenzo[a,h]anthracene (DBA) and six-ring PAHs: benzo[g,h,i]perylene (BghiP), and indeno[1,2,3-cd]pyrene (IP).
